# Serum Lipid Profile and Its Association with Diabetes and Prediabetes in a Rural Bangladeshi Population

**DOI:** 10.3390/ijerph15091944

**Published:** 2018-09-06

**Authors:** Bishwajit Bhowmik, Tasnima Siddiquee, Anindita Mujumder, Faria Afsana, Tareen Ahmed, Ibrahimu A. Mdala, Nayla Cristina do V. Moreira, Abul Kalam Azad Khan, Akhtar Hussain, Gerd Holmboe-Ottesen, Tone Kristin Omsland

**Affiliations:** 1 Department of Community Medicine and Global Health, University of Oslo, 0318 Oslo, Norway; ibramdala@gmail.com (I.A.M.); naylacristinam@yahoo.com.br (N.C.d.V.M.); gerd.holmboe-ottesen@medisin.uio.no (G.H.-O.); t.k.omsland@medisin.uio.no (T.K.O.); 2 Centre for Global Health Research, Diabetic Association of Bangladesh, Dhaka 1000, Bangladesh; tasnimasiddiquee08@yahoo.com (T.S.); president@dab-bd.org (A.K.A.K.); hussain.akhtar@nord.no (A.H.); 3 Department of Pathology, Ibrahim Medical College, Diabetic Association of Bangladesh, Dhaka 1000, Bangladesh; majumderanindita.rpmc@gmail.com; 4 Bangladesh Institute of Research & Rehabilitation of Diabetes, Endocrine and Metabolic Disorders (BIRDEM), Dhaka 1000, Bangladesh; fariaafsana@yahoo.com (F.A.); t.ahmed1963@gmail.com (T.A.); 5 Faculty of Health Science, NORD University, 8049 Bodo, Norway

**Keywords:** lipid profile, diabetes, prediabetes, Bangladesh

## Abstract

Dyslipidemia is commonly associated with diabetes (T2DM). This has been demonstrated for the Caucasian population, but few data are available for Asian Indians. The paper aims to investigate serum lipids (separately or in combination) and their association with glucose intolerance status (T2DM and prediabetes) in a rural Bangladeshi population. A sample of 2293 adults (≥20 years) were included in a community based cross-sectional survey in 2009. Anthropometric measures, blood pressure, blood glucose (fasting and 2-h oral glucose tolerance test) and fasting serum lipids (total cholesterol, T-Chol; triglycerides, Tg; low density lipoprotein cholesterol, LDL-C and high density lipoprotein cholesterol, HDL-C) were registered. Analysis of covariance (ANCOVA) and regression analysis were performed. High Tg levels were seen in 26% to 64% of the participants, depending on glucose tolerance status. Low HDL-C levels were seen in all groups (>90%). Significant linear trends were observed for high T-Chol, high Tg and low HDL-C with increasing glucose intolerance (*p* for trend <0.001). T2DM was significantly associated with high T-Chol (Odds ratio (OR): 2.43, *p* < 0.001), high Tg (OR: 3.91, *p* < 0.001) and low HDL-C (OR: 2.17, *p* = 0.044). Prediabetes showed a significant association with high Tg (OR: 1.96, *p* < 0.001) and low HDL-C (OR: 2.93, *p* = 0.011). Participants with combined high Tg and low HDL-C levels had a 12.75-fold higher OR for T2DM and 4.89 OR for prediabetes. In Asian Indian populations an assessment of serum lipids is warranted not only for T2DM patients, but also for those with prediabetes.

## 1. Introduction

Diabetes (T2DM) and related cardiovascular complications are major public health challenges worldwide. Individuals with T2DM have two- to four-fold increased risk of coronary artery disease (CAD), the leading cause of death among people with T2DM [1]. Dyslipidemia and hypertension are major modifiable risk factors for T2DM and related CAD, which account for more than 87% of disability in low- and middle-income countries [2,3]. Furthermore, prediabetes (an intermediate metabolic state between normoglycemia and T2DM) has also been found to be associated with an increased risk for cardiovascular disease [4].

Lipid abnormalities in patients with diabetes, often termed “diabetic dyslipidemia”, are typically characterized by high total cholesterol (T-Chol), high triglycerides (Tg), low high density lipoprotein cholesterol (HDL-C) and increased levels of small dense LDL particles. Low density lipoprotein cholesterol (LDL-C) levels may be moderately increased or normal. Lipid abnormalities are common in people with T2DM and prediabetes [5,6] but the pattern of the different lipids may vary between ethnic groups, economic levels, and access to health care [7,8]. A recently published meta-analysis reported that abnormal levels of the above-mentioned lipid parameters reflect, to some extent, the risk of T2DM [9]. Furthermore, studies in people with T2DM have found an increased association between CAD and high Tg and low HDL-C combined, compared to the two lipid parameters assessed separately [10,11]. 

Subjects of south Asian background develop diabetes at lower body mass index (BMI) levels and at lower waist circumference (WC) compared with Caucasians [12]. As a result, the World Health Organization (WHO) and International Diabetes Federation (IDF) recommend using specific cut-off points for WC for this group. At similar BMI levels, diabetes prevalence has been identified as higher in Asians compared with Caucasians. These findings may be partly explained by a difference in body fat distribution: South Asians seem to have higher risk of developing visceral adiposity, which is more closely associated with insulin resistance and T2DM than general adiposity [13]. According to the INTERHEART study, Bangladeshis were found to have the highest prevalence of CAD risk factors among the South Asian populations [14]. Like in all other South Asian countries, T2DM and related cardiovascular complications develop 5–10 years earlier in Bangladesh than in western countries, and consequently fatality rates are high among young adults [15,16]. CAD, stroke, and T2DM now account for 36.5%, 18.3%, and 6.7% of deaths, respectively [17]. 

Data on lipid abnormalities are scarce in the Asian Bangladeshi population known to be at high risk for T2DM and CAD. Epidemiological studies have shown an increased prevalence of lipid disorders in both urban and rural populations over time [18,19,20]. Earlier data from the Bangladeshi population living in their home country have shown that dyslipidemia is associated with T2DM and prediabetes and also with hypertension [20,21]. A study of Bangladeshi immigrants conducted in the UK also observed a high level of T-Chol, even higher than in those living in Bangladesh [22], whereas another study of Bangladeshis in the UK observed that high levels of Tg were associated with CAD [23]. Understanding the association between serum lipid patterns and different stages of glucose intolerance is of considerable clinical and public health importance and such data can potentially form the basis for future prevention programs for diabetes and related complications in South Asians. The present paper aims to compare lipid levels and prevalences of lipid abnormalities by glucose tolerance status (normal, prediabetes, T2DM) among subjects in rural Bangladesh and investigate how different serum lipid patterns are associated with prediabetes and T2DM. 

## 2. Subjects and Methods

### 2.1. Study Design and Study Site

Data were obtained from the Chandra Rural Diabetes Study, a population-based cross-sectional study conducted from March to December 2009 [24]. Chandra is a rural community situated 40 km north of Dhaka in Bangladesh. The demographic and social characteristic profile of the general population of Chandra is typically rural. The main livelihood is agriculture and engagement in other agrarian activities. Ten villages were randomly selected from five areas with a total population of approximately 20,000. The survey was conducted in 20 selected spots (e.g., schools, or in front of mosques or houses of village leaders) of those villages. The survey was carried out in two phases; the first phase consisted of a household census of the total population residing in the study locations. Based on the census results, a sample including both men and women was selected in the second phase. 

### 2.2. Sampling Procedure

To determine the required sample size, the formula: n = Z^2^PQ/d^2^ was used, where, Z = 1.96, P for prevalence (DM and impaired glucose regulation) was taken from a previous study in the Chandra area [25], i.e., 0.15; Q = 1 – P, i.e., 0.85, and d = allowable error of known prevalence, i.e., 0.09 × 0.15. Ideally, it should be 0.05 × 0.15; but, to be safe with respect to estimation with a minimum sample size we allowed only 9% (or 0.09) error of prevalence. Thus, the calculated sample size was n = 2687. Around 3000 individuals (both male and female) aged ≥20 years were invited to participate in this study by following a simple randomization procedure from the record of the census list number. During the recruitment period all persons aged ≥20 years, willing to participate and able to communicate were included in the study. Pregnant women and those with a diagnosed acute physical or mental illness were excluded. Among them, 2376 (79.2%) agreed individuals were investigated. A total of 2293 participants (842 men and 1451 women), for whom all the variables were available, were included in the current study.

### 2.3. Collection of Blood Samples

After an overnight fast of 8 h, all the participants were requested to visit a nearby field center. Initially, a sample of 8 mL of venous blood was collected on arrival for fasting glucose (FPG), lipid profiles and insulin measurements. Another 3 mL venous blood was taken 2 h after a 75 g glucose (2hPG) drink. Plasma glucose was measured by the glucose oxidase method using Dimension RxL Max (Siemens AG, Erlangen, Germany). Serum lipids were measured by standard enzymatic procedures (Dimension RxL Max; Siemens AG, Erlangen, Germany). HDL-C was assessed by the direct assay method, and Friedewald’s formula estimated LDL-C. Serum insulin was measured by high performance liquid chromatography (HPLC) based on ion exchange chromatography (Bio-Rad Laboratories, Hercules, CA, USA) and a two-site chemiluminescent immunoassay system (Diagnostic Products Co., Los Angeles, CA, USA), respectively. All biochemical assays were carried out by the same laboratory technician teams using the same methods throughout the study period.

### 2.4. Measurements of Anthropometric Parameters and Blood Pressure

During the 2-h waiting period for OGTT, a pretested questionnaire was administered to obtain socio-demographic, anthropometric and clinical information. After completion of the interview, trained field workers did anthropometric measurements of the participants dressed in light clothing and bare feet. Body weight was measured to the nearest 0.1 kg using a digital scale. BMI was calculated using the height and weight measurement (kg/m^2^). WC was measured to the nearest 0.1 cm by placing a plastic tape at the midpoint between the lower rib margin and the iliac crest. Hip circumference was measured at the greatest protrusion of the buttocks. A ten-minute rest was assured before measurement of blood pressure and a standard adult cuff was used to minimize variation in measurement. Blood pressure was measured twice in the right arm in both sitting and standing position. Measurements were taken 5 min apart, and the mean of the two measurements was taken as the final blood pressure reading. 

### 2.5. Definition of Variables

Obesity for both sexes was defined as BMI of ≥ 25 kg/m^2^; central obesity including WC for male and female were ≥90 cm and ≥80 cm, respectively [26,27]. T2DM was defined as FPG ≥ 7.0 mmol/L or 2hPG ≥ 11.1 mmol/L. Prediabetes was defined as FPG ≥ 6.1 mmol/L to <7.0 mmol/L (impaired fasting glycemia) and 2hPG ≥ 7.8 mmol/L to <11.1 mmol/L (impaired glucose tolerance). Normal glucose tolerance (NGT) was defined as FPG <6.1 mmol/L and 2hPG <7.8 mmol/L [28]. Hypertension (HTN) was defined as systolic blood pressure (SBP) ≥ 140 mmHg and diastolic blood pressure (DBP) ≥ 90 mmHg or already on anti-hypertensive medication (s) or told to have HTN by a physician [29]. Cut-off values for serum lipid profiles were: high T-Chol ≥ 5.0 mmol/L, high Tg ≥ 1.7 mmol/L, high LDL-C ≥ 3.4 mmol/L, and low HDL-C <1.04 mmol/L (for men) and <1.3 mmol/L (for women) [30]. Using the above-mentioned cut points of Tg and HDL-C, we also divided the subjects’ Tg and HDL-C status into the following four categories: 0 = “normal Tg and normal HDL-C” (reference category); 1 = “High Tg and normal HDL-C”; 2 = “normal Tg and low HDL-C”; and 3 = “high Tg and low HDL-C” [10]. HOMA-IR (homeostatic model assessment for insulin resistance) was calculated by using the method of Matthews et al. (fasting serum insulin in µU/mL × FPG in mmol/L)/22.5 [31].

### 2.6. Ethical Approval

The protocol was approved by Ethical Review Committee of Diabetic Association of Bangladesh (7 February 2009). Research participation, confidentiality, and consent were followed as per Helsinki declaration, with local adaptation to allow both verbal and written instructions. Ethical approval was not needed in Norway as an anonymous data file was used for the analyses. 

### 2.7. Statistical Analyses

Continuous variables were expressed by means and 95% confidence intervals (CIs) adjusted for age and percentages and 95% CIs expressed categorical variables. Skewed data (including Tg, fasting insulin, HOMA-IR) were log-transformed before analysis, and the results were transformed back to the original scale. Analysis of covariance (ANCOVA) tested differences between the two groups of means adjusted for age, and pairwise comparisons between the groups were performed and corrected for multiple testing using Bonferroni method. Logistic regression models were used to examine the statistical difference of proportions and for estimates of odds ratios adjusted for age. A trend analysis test was used to determine the differences in proportions and means across the groups. Multiple logistic regression analysis adjusted for age was used to estimate the association of lipid parameters including T-Chol, Tg, HDL-C, and LDL-C with T2DM and prediabetes. Both unadjusted and adjusted (for age, central obesity, and HTN) logistic regression analyses were used to estimate the association of high Tg and low HDL with T2DM and prediabetes. 

PASW statistics version 21 for Windows (SPSS Inc., Chicago, IL, USA), STATA 14 for Windows (STATA Co., College Station, TX, USA), and Medcalc software were used as needed. Statistical inference was based on 95% confidence intervals (CIs), and the significance level was set at 0.05. 

## 3. Results

The characteristics of the study participants based on categories of glucose intolerance status are displayed in Table 1. Participants with prediabetes and T2DM were older, more obese (both general and central obesity) and were more hypertensive than the NGT group. 

Adjusted means of FPG, 2hPG, fasting insulin, HOMA-IR, T-Chol, Tg, and HDL-C levels varied significantly between the different stages of glucose intolerance (Table 1). Except for the HDL-C which was negatively associated with the level of glucose intolerance, positive linear trends were observed (*p*-value for trend <0.001) between these risk variables and the increasing levels of glucose intolerance. Low HDL-C levels were common (>90%) in all groups of glucose intolerance. The prevalence of high Tg varied from 26.1% in those with NGT to 63.5% in those with T2DM (Table 1). 

The proportions having high T-Chol, high Tg, low HDL-C, combined high Tg and low HDL-C were significantly higher among participants with T2DM and prediabetes than those with NGT. In participants with NGT, 24.5% had high Tg combined with low HDL-C, whereas the corresponding prevalence in those with T2DM was 58.7%. 

**Figure 1:**
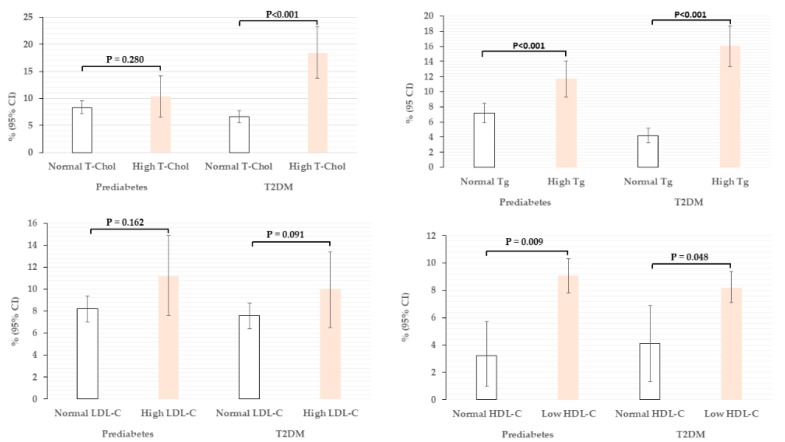
Prevalence of prediabetes and T2DM by different lipid parameters.

The proportion of participants with T2DM and prediabetes by different lipid parameters are displayed in Figure 1. The proportion of T2DM among those with normal or high T-Chol; normal or high Tg; normal or high LDL-C; and normal or low HDL-C groups were: 6.6 vs. 18.4% (*p* < 0.001); 4.2 vs. 16.1% (*p* < 0.001); 7.6 vs. 10.0% (*p* = 0.162); and 4.1 vs. 8.2% (*p* = 0.048), respectively. 

The proportion of prediabetes among those with normal or high T-Chol; normal or high Tg; normal or high LDL-C; and normal or low HDL-C groups were: 8.3 vs. 10.4% (*p* = 0.280); 7.2 vs. 11.7% (*p* = 0.001); 8.1 vs. 11.2% (*p* = 0.091); and 3.2 vs. 9.1% (*p* = 0.009), respectively.

No significant differences were observed in the proportion of T2DM and prediabetes between high or normal LDL-C groups. 

Data are presented as percentages (95% confidence interval) adjusted for age. Independent variables were categorized as follows: T-Chol (normal < 5.2 vs. high ≥ 5.2 mmol/L); Tg (normal < 1.7 vs. high ≥ 1.7 mmol/L); HDL-C (normal M ≥ 1.04 and F ≥ 1.3 vs. Low M <1.04 and F < 1.3 mmol/L); LDL-C (normal < 3.4 vs. high ≥ 3.4 mmol/L). Abbreviation: T2DM, type 2 diabetes mellitus; T-Chol, total cholesterol; Tg, triglycerides; LDL-C, low density lipoprotein cholesterol; HDL-C, high density lipoprotein cholesterol. 

Table 2 shows the odds ratio (OR) of different lipid parameters for the risk of having T2DM and prediabetes. T2DM showed a significant association with T-Chol (OR): 2.43, *p* < 0.001); Tg (OR: 3.91, *p* < 0.001); and HDL-C (OR: 2.17, *p* = 0.044). Prediabetes showed a significant association with Tg (OR: 1.96, *p* < 0.001) and HDL-C (OR: 2.93, *p* = 0.011). 

Table 3 shows the associations of combined Tg and HDL-C status according to different combinations of high/low categories of these two lipids among those with T2DM and prediabetes. The proportion of T2DM among normal Tg and normal HDL-C; high Tg and normal HDL-C; normal Tg and low HDL-C; and high Tg and low HDL-C groups were: 1.4%, 15.0%, 4.4% and 16.6%, respectively. The proportion of prediabetes among normal Tg and normal HDL-C; high Tg and normal HDL-C; normal Tg and low HDL-C; and high Tg and low HDL-C groups were: 2.9%, 5.0%, 7.6%, and 12.3%, respectively. After adjustments for age, hypertension and central obesity subjects with high Tg and low HDL-C levels had a 12.75-fold greater OR for T2DM and 4.89-fold greater OR for prediabetes than those with normal Tg and normal HDL-C levels. The corresponding adjusted OR for those with high Tg and normal HDL-C levels was 10.47. 

## 4. Discussion

To the best of our knowledge, the present study is one of the few studies of a South Asian population attempting to assess the association between serum lipids (including T-Chol, Tg, LDL-C, and HDL-C) and degrees of glucose intolerance. We found the levels of dyslipidemia, especially high Tg and low HDL-C, alarmingly high; more than 90% had low HDL-C levels and the prevalence of high Tg varied from 26% among those with NGT to 64% among those with T2DM. Furthermore, our results showed a strong association between serum lipids and T2DM and prediabetes. Significant linear trends for glucose tolerance status were observed for high T-Chol, high Tg and low HDL-C. In addition, high levels of Tg in combination with low levels of HDL-C showed the highest association with T2DM and prediabetes. Whereas the levels of high T-Chol, high Tg, and low HDL-C were more elevated among participants with T2DM and prediabetes, the levels of LDL-C did not differ significantly between the glycemic groups. 

As in the current study, high prevalences of high Tg (up to 70%) have been reported for South Asians [32,33]. Low HDL-C levels are also common in South Asians of whom about one third has been found to have low HDL-C levels, but our result shows a prevalence that is almost three times as high. However, the cardiovascular protection of HDL-C in South Asians appears to be smaller compared to other ethnic groups [34]. Whether low HDL is a true risk factor for increased cardiovascular risk in South Asians is not known, and new studies are needed to investigate this further. 

Our findings are mainly in agreement with two landmark studies namely the Framingham Heart Study [35] and the UK Prospective Diabetes Study (UKPDS) [36]. In both studies T2DM subjects compared to those without T2DM, had higher plasma Tg levels and lower HDL-C levels. However, T-Chol level was found significantly increased in female diabetic subjects in the Framingham Heart Study. The LDL-C level in subjects with glucose intolerance did not differ from their non-diabetic counterparts in neither of the studies. Moreover, results from the pan-European Survey, USA, China, and India are also in agreement with our findings regarding high Tg and low HDL-C levels in prediabetic subjects [37,38,39,40]. Our study also observed that the proportion with T2DM was significantly higher in subjects with high T-Chol, high Tg, and low HDL-C groups, whereas, prediabetes was higher in those with high Tg and low HDL-C groups. 

The Strong Heart study aimed at investigating if combined high Tg and low HDL-C status, also known as “atherogenic dyslipidemia”, were more likely to be present in T2DM individuals [10]. This study, based on a prospective cohort, showed that high fasting Tg level in combination with a low HDL-C level were associated with increased risks of CAD and ischemic stroke, particularly in those with diabetes. It was further shown that 60% of the participants with combined high TG and low HDL levels had T2DM, whereas the corresponding figure for non-diabetics was 30%. In our study, high Tg was also strongly associated with T2DM even when HDL-C was normal. Participants with combined high Tg and low HDL-C levels had an estimated 13-fold greater odds of T2DM and estimated five-fold greater odds of prediabetes than those with normal Tg and normal HDL-C levels.

It is recognized that dyslipidemia is an independent risk factor for cardiovascular disease. Elevated blood glucose level combined with dyslipidemia increases atherosclerosis-related inflammation and makes it more extensive [41]. A larger extent of coronary artery calcification in asymptomatic patients with newly-diagnosed T2DM has been demonstrated [42]. Dyslipidemia is not only an important risk for macrovascular complications [43]; studies have also observed the association of dyslipidemia with microvascular complications related to T2DM namely diabetic retinopathy, diabetic nephropathy and diabetic neuropathy [44,45,46].

Several factors are related to diabetic dyslipidemia including insulin effects on liver apoprotein production, regulation of lipoprotein lipase, actions of cholesteryl ester transfer protein (CETP), and peripheral actions of insulin on adipose and muscle tissue [47]. The process for the development of cardiac complication is based on the dyslipidemia- insulin resistance (IR)–hyperinsulinemia cycle, well known as the “vicious cycle hypothesis” [48]. In an insulin-resistant state, hypertriglyceridemia is primarily due to an increased hepatic production of very low density lipoprotein (VLDL) particles, postprandial hyperlipidemia, and low lipoprotein lipase (LPL) levels. This hypertriglyceridemia enhances the CETP mediated interchange of Tg from Tg-rich lipoproteins to HDL-L/HDL-VL and the subsequent Tg-enrichment of HDL-C. Hepatic lipase has greater activity against Tg and will, thus, convert large HDL particles to small HDL particles, which are also cleared more rapidly from the circulation by the kidney, consequently reducing the concentration of HDL particles (HDL-P) [49,50]. All the markers of IR, including fasting insulin, HOMA-IR, BMI, WC, SBP, FPG levels were significantly higher in subjects with T2DM and prediabetes compared to NGT subjects in our study population. Increased levels of all these IR markers suggest that our study population is at high risk for atherosclerosis, contributing to an increased incidence of T2DM and CAD in our country.

The strengths of the current study include a population-based study design with random sampling, a reasonably large sample size, and high participation rate. As demographics and living conditions in Chandra are similar to a majority of the rural Bangladeshi population, the result may be generalized to most of the rural population in Bangladesh which constitutes about 70% of the total population. Serum lipids measures were obtained in a fasting state; thus, interpretation of the study results can be relevant in primary care practice in Bangladesh.

A limitation of the present study is that it is cross-sectional and, thus, cannot determine causality. However, this type of study might very well identify important associations which can be further investigated in future studies. Hence, the findings of this study will help to provide a baseline for future studies in Asian Indian populations to examine relationships between lipid disorders and the risk of DM or CAD. Other potential factors such as dietary habits, physical activity level, smoking habit, medication (s) and concomitant diseases influencing lipid levels were not evaluated. Moreover, only the routine lipid parameters were assessed in the present study. Other important lipid measures, such as the number of LDL particles (rather than the cholesterol content in LDL), HDL functionality, Sphingosine-1-phosphate (S1P) content of the HDL particle and LDL particles, which are more predictive of cardiometabolic risk and more characteristic of diabetic dyslipemia [51,52,53], could have been interesting to obtain to increase our understanding.

### Clinical Implications

This is one of few studies, which explores the association of routine lipid parameters with T2DM and prediabetes in Bangladesh. Low HDL-C and high Tg dyslipidemia are more prevalent in our population than in the other South Asian populations [8,54] and have shown stronger association with T2DM and prediabetes. In clinical practice in Bangladesh, T-Chol, Tg, HDL-C, and LDL-C are the parameters available for monitoring lipid abnormalities. However, as in most developing countries the cost of such measures are high and the access to laboratory measurements is limited. At present, there is no evidence-based recommendation for screening of lipid disorders in most developing countries including Bangladesh, even though cardiovascular complications (many of which are related to lipid disorders) have been shown to be significant contributors directly or indirectly to the costs of diabetes care [55].

Laboratory testing and statistically defined criteria are needed to assess lipid disorders. Although the benefits of screening and treatment of lipid disorders in people with known cardiovascular diseases are recognized, controversy remains regarding screening of asymptomatic individuals who are not known to be at increased risk of CAD or T2DM. Since 2004, the American Diabetes Association (ADA) has recommended screening of high-risk adults at any age for diabetes who have high BMI (≥25 kg/m^2^), low HDL-C (<0.90 mmol), and or a high Tg level (>2.82 mmol/L) [56]. Studies suggest that under-diagnosis and under-treatment of lipid disorders result in higher rates of diabetes-related microvascular and macrovascular complications, such as myocardial infarction, stroke, nephropathy, and retinopathy in the Asian Indian population [44,57]. A systematic review and meta-analysis of randomized controlled trials (RCTs) have clearly shown that T2DM patients benefit more from treatment with lipid lowering drugs than do non-diabetic patients [58]. Therefore, early screening and correction of lipid disorders are highly recommended for the primary and secondary care prevention of T2DM.

## 5. Conclusions

In this rural Bangladeshi population, the prevalences of dyslipidemia, especially low HDL-C and high Tg were found to be alarmingly high. The proportion of subjects with unfavorable lipid profiles increased with degree of glucose intolerance. High levels of Tg in combination with low levels of HDL-C showed the strongest association with T2DM and prediabetes. This paper suggests that routine monitoring of the commonly used lipid parameters (especially Tg and HDL-C) among patients with T2DM and prediabetes, is warranted in this population considered to be the epi-center for T2DM or CAD.

## Figures and Tables

**Table 1:** Characteristics of the study participants by the level of glucose intolerance.

Variable	Normal (n = 1915)	Prediabetes (n = 197)	T2DM (n = 181)	*p* for Trend
Age (years)	41.2 (40.6, 41.8)	44.2 (42.3, 46.1) *	45.8 (43.8, 47.7) *	<0.001
Female, %	64.6 (49.9, 63.8)	56.9 (50.0, 63.8)	55.8 (48.5, 63.1)	0.003
Body Mass Index (kg/m^2^)	22.3 (22.2, 22.5)	24.3 (23.7, 24.8) *	24.4 (23.8, 24.9) *	<0.001
BMI (≥25 kg/m^2^), %	23.1 (21.2, 24.9)	43.2 (36.3, 50.1)*	41.4 (34.2, 48.6) *	<0.001
Waist (cm)	79.4 (78.9, 79.9)	84.8 (83.3, 86.4) *	86.9 (85.5, 88.3) *	<0.001
Waist: M ≥ 90 & F ≥ 80 cm, %	36.1 (33.9, 38.1)	56.4 (49.7, 63.0) *	62.1 (55.4, 68.9) *^,†^	<0.001
SBP (mmHg)	115.3 (114.6, 116.0)	118.3 (116.6, 120.0) *	120.9 (1191, 122.6) *^,†^	<0.001
DBP (mmHg)	76.6 (76.1, 77.0)	78.0 (77.0, 79.1) *	79.4 (78.3, 80.5) *	<0.001
Hypertension, %	14.4 (12.8, 15.9)	17.0 (12.0, 21.9)	24.8 (18.9, 30.7) *^,†^	<0.001
FPG (mmol/L)	4.7 (4.6, 4.8)	5.7 (5.5, 5.9) *	9.5 (9.3, 9.7) *^,†^	<0.001
2hPG (mmol/L)	5.4 (5.3, 5.5)	7.5 (7.4, 7.9) *	13.9 (13.8, 14.2) *^,†^	<0.001
Fasting insulin (µIu/mL) ^±^	7.9 (7.8, 8.2)	10.5 (9.4, 11.6) *	11.5 (10.5, 12.5) *	<0.001
HOMA-IR ^±^	1.50 (1.45, 1.54)	2.36 (2.12, 2.64) *	4.14 (3.67, 4.71) *^,†^	<0.001
T-Chol (mmol/L)	4.3 (4.2, 4.4)	4.5 (4.4, 4.6) *	4.9 (4.7, 5.0) *^,†^	<0.001
T-Chol ≥ 5.2 mmol/L, %	8.7 (7.5, 10.0)	13.2 (9.1, 18.7) *	26.0 (20.1, 32.9) *^,†^	<0.001
Tg (mmol/L) ^±^	1.3 (1.1, 1.4)	1.5 (1.3, 1.6) *	1.9 (1.8, 2.1) *^,†^	<0.001
Tg ≥ 1.7 mmol/L, %	26.1 (24.2, 28.1)	42.1 (35.4, 49.2) *	63.5 (56.3, 70.2) *^,†^	<0.001
HDL-C (mmol/L)	0.91 (0.90, 0.92)	0.86 (0.82, 0.89) *	0.81 (0.77, 0.85) *^,†^	<0.001
HDL-C: M:<1.04 & F:<1.3 mmol/L, %	91.3 (90.0, 92.5)	97.0 (93.4, 99.0) *	95.6 (91.4, 97.8) *	0.004
LDL-C (mmol/L)	2.76 (2.72, 2.78)	2.80 (2.70, 2.89)	2.84 (2.75, 2.92)	0.016
LDL-C ≥ 3.4 mmol/L, %	11.3 (9.9, 12.8)	16.2 (11.7, 22.1) *	16.0 (11.4, 22.1)	0.014
High Tg & Low HDL-C, %	24.6 (22.7, 26.6)	40.0 (33.2, 46.7) *	58.7 (51.5, 65.9) *^,†^	<0.001

Data are means (95% confidence interval) or percentages (95% confidence interval) adjusted for age as indicated. ^±^ Geometric means (95% confidence interval) for Tg, fasting insulin and HOMA-IR. * *p*-values < 0.05 compared with normal; ^†^ *p*-values < 0.05 compared with pre-diabetes. Abbreviation: T2DM, type 2 diabetes; BMI, body mass index; SBP, systolic blood pressure; DBP, diastolic blood pressure; FPG, fasting plasma glucose; 2hPG, 2 h plasma glucose; HOMA-IR, homeostatic model assessment for insulin resistance); T-Chol, total cholesterol; Tg, triglycerides; HDL-C, high-density lipoprotein cholesterol; LDL-C, low-density lipoprotein cholesterol.

**Table 2:** Odds ratio (OR) with 95% CI of different lipid parameters for having risk of T2DM and prediabetes.

Lipid Parameters	Prediabetes		T2DM	
	OR (95% CI)	*p* Value	OR (95% CI)	*p* Value
T-Chol	0.90 (0.50, 1.61)	0.731	2.43 (1.46, 4.04)	<0.001
Tg	1.96 (1.42, 2.69)	<0.001	3.91 (2.78, 5.51)	<0.001
HDL-Cholesterol	2.93 (1.27, 6.73)	0.011	2.17 (1.02, 4.61)	0.044
LDL-Cholesterol	1.40 (0.84, 2.33)	0.193	0.63 (0.36, 1.11)	0.109

Multiple logistic regression analysis adjusted for age was applied to generate OR and 95% CI. Independent variables were categories as follows: Cholesterol (normal < 5.2 vs. high ≥ 5.2 mmol/L); Triglycerides (normal < 1.7 vs. high ≥ 1.7 mmol/L); HDL-C (normal M ≥ 1.04 & F ≥ 1.3 vs. Low M < 1.04 and F < 1.3 mmol/L); LDL-C (normal < 3.4 vs. high ≥ 3.4 mmol/L). Abbreviation: T2DM, type 2 diabetes mellitus; CI, confidence interval; T-Chol, total cholesterol; Tg, triglycerides; HDL-C, high-density lipoprotein; LDL-C, low-density lipoprotein.

**Table 3:** Associations of combined Tg and HDL-C status according to different combinations of high/low categories of these two lipids among those with T2DM (n = 181) and prediabetes (n = 197).

	N (%)		Unadjusted		Adjusted	
T2DM	Cases	Events	OR (95% CI)	*p* Value	OR (95% CI)	*p* Value
Normal Tg and Normal HDL-C	140 (6.1)	2 (1.4)	Reference		Reference	
High Tg and Normal HDL-C	40 (1.7)	6 (15.0)	12.56 (2.42, 65.15)	0.003	10.47 (1.99, 55.17)	0.006
Normal Tg and Low HDL-C	1455 (63.5)	64 (4.4)	3.35 (0.81, 13.82)	0.095	3.31 (0.80, 13.74)	0.099
High Tg and Low HDL-C	658 (28.7)	109 (16.6)	15.60 (3.80, 64.02)	<0.001	12.75 (3.08, 52.65)	<0.001
**Prediabetes**						
Normal Tg and Normal HDL-C	140 (6.1)	4 (2.9)	Reference		Reference	
High Tg and Normal HDL-C	40 (1.7)	2 (5.0)	2.09 (0.37, 11.94)	0.405	1.95 (0.34, 11.20)	0.453
Normal Tg and Low HDL-C	1455 (63.5)	110 (7.6)	2.88 (1.04, 7.92)	0.041	2.81 (1.02, 7.77)	0.046
High Tg and Low HDL-C	658 (28.7)	81 (12.3)	5.80 (2.09, 16.11)	0.001	4.89 (1.75, 13.68)	0.002

Adjusted for age, hypertension, and central obesity. Variables were categorized as follows: Tg (normal < 1.7 vs. high ≥ 1.7 mmol/L); HDL-C (normal M ≥ 1.04 & F ≥ 1.3 vs. low M < 1.04 and F < 1.3 mmol/L). Abbreviation: T2DM, type 2 diabetes mellitus; CI, confidence interval; Tg, triglycerides; HDL-C, high-density lipoprotein.
